# Improved YOLOX-Tiny network for detection of tobacco brown spot disease

**DOI:** 10.3389/fpls.2023.1135105

**Published:** 2023-02-14

**Authors:** Jianwu Lin, Dianzhi Yu, Renyong Pan, Jitong Cai, Jiaming Liu, Licai Zhang, Xingtian Wen, Xishun Peng, Tomislav Cernava, Safa Oufensou, Quirico Migheli, Xiaoyulong Chen, Xin Zhang

**Affiliations:** ^1^ College of Big Data and Information Engineering, Guizhou University, Guiyang, China; ^2^ Guizhou-Europe Environmental Biotechnology and Agricultural Informatics Oversea Innovation Center in Guizhou University, Guizhou Provincial Science and Technology Department, Guiyang, China; ^3^ International Jointed Institute of Plant Microbial Ecology and Resource Management in Guizhou University, Ministry of Agriculture, China Association of Agricultural Science Societies, Guiyang, China; ^4^ Institute of Environmental Biotechnology, Graz University of Technology, Graz, Austria; ^5^ Dipartimento di Agraria and Nucleo di Ricerca sulla Desertificazione - NRD, Università degli Studi di Sassari, Sassari, Italy

**Keywords:** object detection, tobacco brown spot disease, YOLOX-Tiny network, hierarchical mixed-scale units, convolutional block attention modules

## Abstract

**Introduction:**

Tobacco brown spot disease caused by *Alternaria* fungal species is a major threat to tobacco growth and yield. Thus, accurate and rapid detection of tobacco brown spot disease is vital for disease prevention and chemical pesticide inputs.

**Methods:**

Here, we propose an improved YOLOX-Tiny network, named YOLO-Tobacco, for the detection of tobacco brown spot disease under open-field scenarios. Aiming to excavate valuable disease features and enhance the integration of different levels of features, thereby improving the ability to detect dense disease spots at different scales, we introduced hierarchical mixed-scale units (HMUs) in the neck network for information interaction and feature refinement between channels. Furthermore, in order to enhance the detection of small disease spots and the robustness of the network, we also introduced convolutional block attention modules (CBAMs) into the neck network.

**Results:**

As a result, the YOLO-Tobacco network achieved an average precision (AP) of 80.56% on the test set. The AP was 3.22%, 8.99%, and 12.03% higher than that obtained by the classic lightweight detection networks YOLOX-Tiny network, YOLOv5-S network, and YOLOv4-Tiny network, respectively. In addition, the YOLO-Tobacco network also had a fast detection speed of 69 frames per second (FPS).

**Discussion:**

Therefore, the YOLO-Tobacco network satisfies both the advantages of high detection accuracy and fast detection speed. It will likely have a positive impact on early monitoring, disease control, and quality assessment in diseased tobacco plants.

## 1 Introduction

Tobacco (*Nicotiana tabacum* L.) is an economically important crop in China. Although it is well known “smoking is not good for heathy”, the plant cultivation was dominant in some areas as one of the main income sources for local farmers (Chen et al., 2020). In addition, tobacco is a model plant in biotechnology research, as well as a provider of secondary metabolites that could be used by human being. For instance, Nicotine is widely used in pharmaceutical industry, as well as pesticide innovation (Chen et al., 2021). Tobacco production is affected by various diseases that limit yields and product quality. For example, tobacco brown spot disease (Xie et al., 2021) caused by *Alternaria* fungal species is widely distributed and frequent in China, where it causes heavy economic losses. Timely detection and prevention of the disease provide effective means to solve the problem (Lin et al., 2022a). The traditional crop disease detection method mainly relies on hand-designed features. The detection efficiency and detection accuracy of this method are low, which can no longer meet the needs of modern agriculture (Singh and Misra, 2017; Lin et al., 2022b).

With the rapid development of deep learning, object detection techniques based on deep learning are widely used in computer vision. Girshick et al. (2014) combined region proposal and convolutional neural networks (CNNs) to design the first two-stage network Regions with CNN features (R-CNN). Some researchers improved R-CNN, and a faster and more accurate network called Fast R-CNN (Girshick, 2015) was proposed. Subsequently, Ren et al. (2015) proposed the Faster-RCNN network based on Fast-RCNN, which was the first detection network to implement end-to-end. At present, single-stage detection networks, such as single shot multibox detector (SSD) (Liu et al., 2016), RetinaNet (Lin et al., 2017), and you only look once (YOLO) series (Redmon et al., 2016; Redmon and Farhadi, 2017; Redmon and Farhadi, 2018; Bochkovskiy et al., 2020; Ge et al., 2021), are more widely used because they have faster detection speed than two-stage detection networks. With the deep integration between deep learning and agricultural production, smart agriculture has become a major trend in the development of modern agriculture in different countries (Kamilaris and Prenafeta-Boldú, 2018; Nguyen et al., 2020). The use of cameras mounted on hardware devices to determine whether leaves are infected by pathogens has been widely used in the field of smart agriculture, leading to the automatic identification of crop diseases (Kulkarni et al., 2021; Gajjar et al., 2022). In recent years, increasing studies were focused in this field, Li et al. (2022) developed an improved YOLOv5-S network to detect five vegetable diseases. The experimental results showed that the mean average precision (mAP) of the improved YOLOv5-S network reached 93.1%, which was higher than nanodet-plus, YOLOv4-Tiny, and YOLOX-S. Wang et al. (2021) proposed a YOLOv3-Tiny-IRB based on the YOLOv3-tiny network architecture for detecting tomato diseases and pests. The experimental results showed that the mAP under three conditions: (a) deep separation, (b) debris occlusion, and (c) leaves overlapping reached 98.3%, 92.1%, and 90.2%, respectively. Zhao and Qu (2018) used the YOLOv2 algorithm to detect healthy and diseased tomatoes with a mAP of 91%. Qi et al. (2022) utilized an improved SE-YOLOv5 to recognize tomato virus disease: the mAP achieved 94.1%, which was 1.23%, 16.77%, and 1.78% higher than that of the Faster R-CNN model, SSD model, and YOLOv5 model, respectively. Fuentes et al. (2019) proposed an improved Faster R-CNN algorithm, which can effectively detect and locate plant abnormalities. An average accuracy of 92.5% was achieved in the built tomato plant abnormality description dataset. Zhang et al. (2022) combined the YOLOv5 network with distance intersection over union non maximum suppression (DIOU-NMS) to detect and record wheat ears in images collected from field plots. In addition, they also used HSV and CMYK color space to extract comprehensive color feature (CCF) and used the Res-Net network to extract each wheat ear’s high dimension feature. The average accuracies of counting total wheat ears and diseased wheat ears were 96.16% and 81.66%, respectively. Chen et al. (2022) proposed an improved YOLOv5 using a new involution bottleneck module and SE modules for plant disease recognition, The test results showed that the mAP of the improved YOLOv5 network reached 70%. He et al. (2021) proposed an improved SSD network for the detection of watermelon diseases. Experiments showed that the average accuracy of the improved SSD network was 92.4%. Moreover, Bao et al. (2022) proposed an improved RetinaNet network, named AX-RetinaNet, for the automatic detection and identification of tea leaf diseases in natural scene images.

In general, the findings of the abovementioned study confirm that object detection technology has may advantages in crop disease detection, leading to prompt adoption of targeted control measures. The objective of this study was to examine tobacco brown spot disease in real scenes with complex backgrounds. Images of tobacco brown spot disease collected in natural conditions have three characteristics. Usually, the distribution of spots is too dense and inconsistent in size, the symptoms of some spots are not obvious, and the light distribution is uneven in some images. Therefore, existing object detection networks do not meet the demand for accurate and fast detection of tobacco brown spot disease in natural environments. Hence, we propose an improved detection network, named YOLO-Tobacco, for the detection of tobacco brown spot diseases. The main aims of this study are as follows:

(1) An improved detection network named YOLO-Tobacco is proposed for the detection of tobacco brown spot diseases under natural conditions based on the YOLOX-Tiny network.
(2) To facilitate the effective fusion between different levels of features, thus enhancing the detection of dense disease spots at different scales, the hierarchical mixed-scale units (HMUs) (Pang et al., 2022) were introduced in the neck network to refine the critical disease features.
(3) To further enhance the ability to extract useful features, thereby improving the robustness and the ability to detect small objects of the model, the convolutional block attention modules (CBAMs) (Woo et al., 2018) were implemented in the neck network.

The rest of the study is organized as follows: Section 2 introduces the collection and preprocessing of the dataset. Then, Section 3 introduces the proposed method. Subsequently, experimental results and analysis are present in Section 4. Lastly, the conclusion is summarized in Section 5.

## 2 Materials

### 2.1 Image acquisition

**Figure 1:**
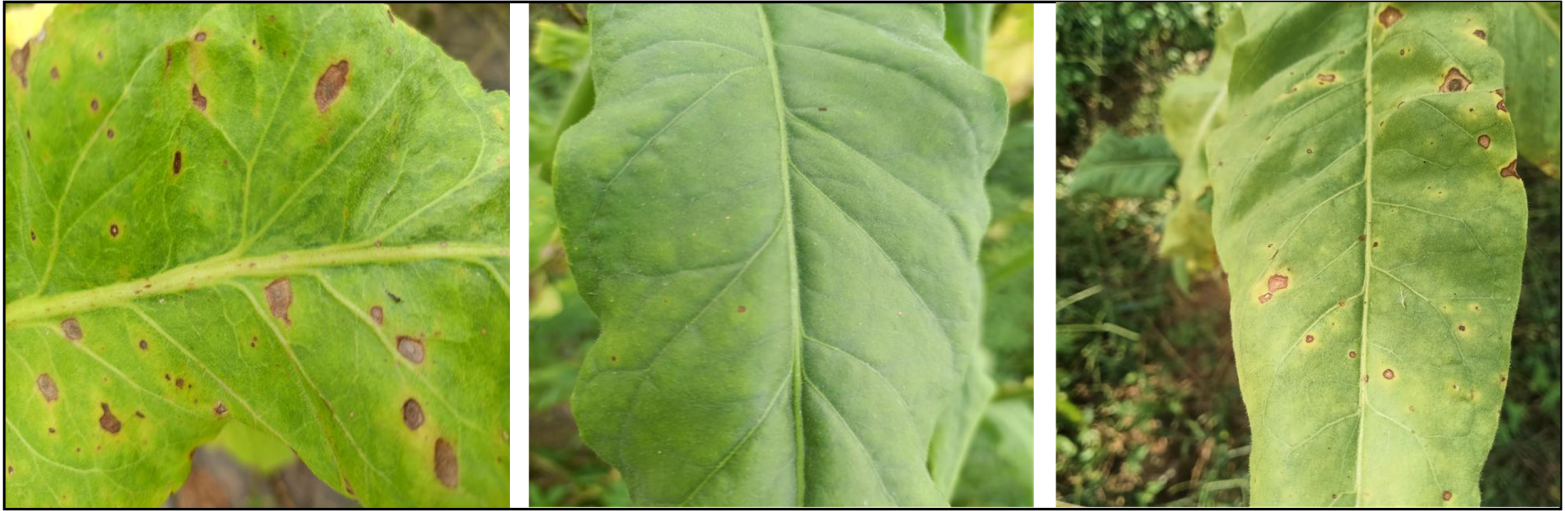
Examples of material that was collected for the dataset. Images of leaves of the tobacco plant (*Nicotiana tabacum* L.) affected by tobacco brown spot disease were used to compile the dataset that was used in this study.

We obtained tobacco leaf datasets from a tobacco research demonstration site in Sinan County, Tong Ren City, Guizhou Province, China. The period of image acquisition was from October 1 to October 7, 2021. The device used for image acquisition was an iPhone 8 plus device. We collected a total of 340 images of tobacco brown spot disease caused by *Alternaria alternata*. Representative images are shown in **Figure 1**.

### 2.2 Image preprocessing

**Figure 2:**
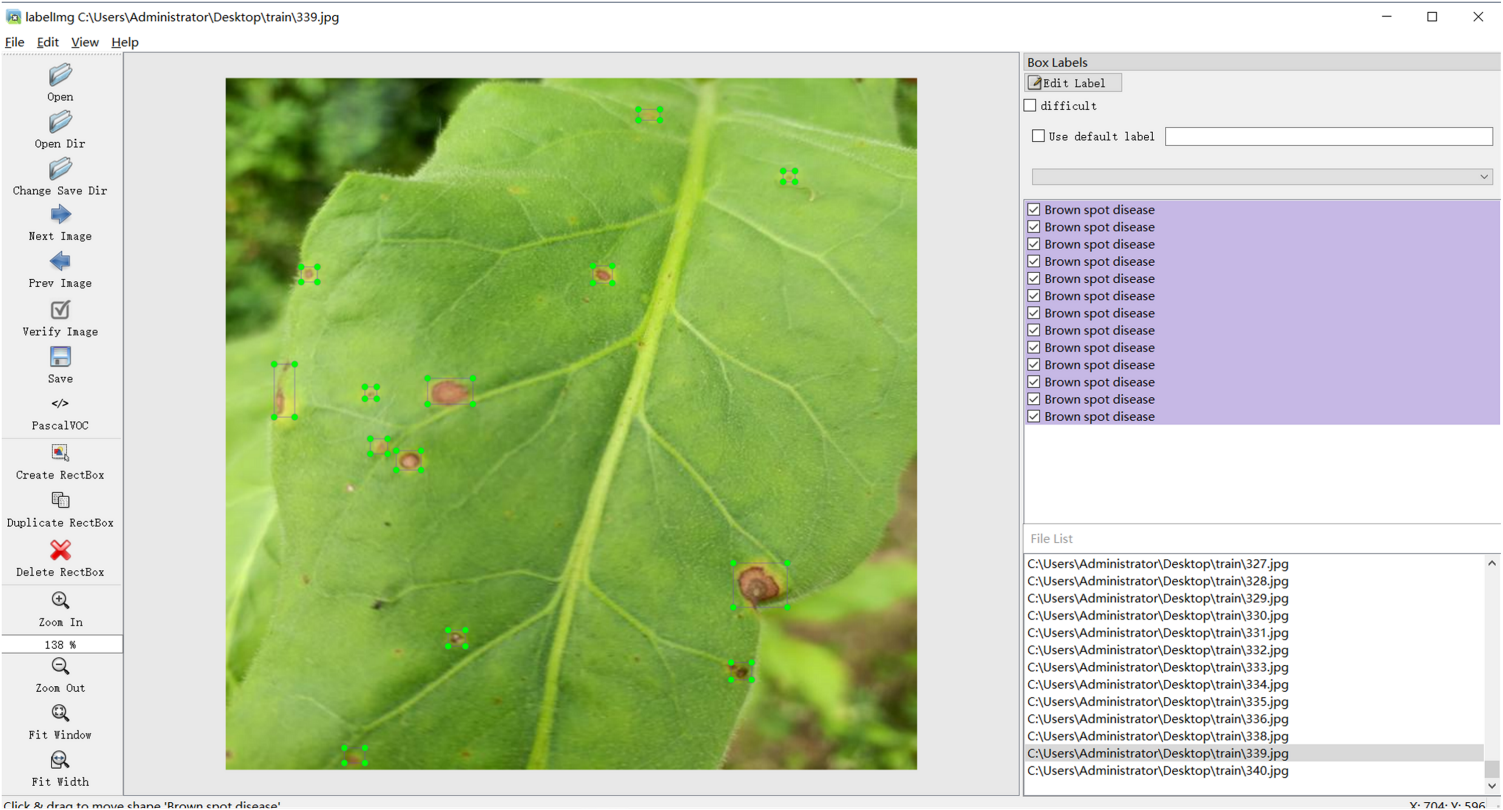
Process of tobacco brown spot disease symptom labeling with LabelImg.

We used LabelImg (Tzutalin, 2015) to label 340 images of tobacco brown spot disease. The process of labeling is shown in **Figure 2**. Owing to the time, equipment, and location of the image acquisition site, the number of tobacco brown spot disease images was limited. Thus, the dataset had to be augmented to increase the diversity of training samples, reduce model overfitting, and improve the generalization ability of the network. To ensure that the distributions of the training set and test set are independent, the following operations were carried out: firstly, the tobacco brown spot disease datasets were divided into a training set and a test set in the ratio of 8:2; then, the training set was augmented using rotation, brightness enhancement, adding of Gaussian noise enhancement, and color enhancement, while the test set was not augmented. The specific data distribution is shown in **Table 1**.

**Table 1:** Sample distribution.

Dataset	Training set	Test set
Original images	272	68
Rotation 60	272	0
Rotation 90	272	0
Rotation 180	272	0
Rotation 270	272	0
Brightness enhancement	272	0
Add gaussian noise enhancement	272	0
Color enhancement	272	0
Total images	2176	68

## 3 Proposed method

### 3.1 YOLO-Tobacco network framework

YOLOX is one of the newest models in the YOLO series, which consists of seven sub-models, YOLOX-Nano, YOLOX-Tiny, YOLOX-S, YOLOX-L, and YOLOX-M. YOLOX-Tiny achieves a trade-off between detection accuracy and detection speed. The number of parameters of YOLOX-Tiny only accounts for 5.06 million, which is suitable for deployment on various agricultural hardware devices. However, for the detection of dense small disease spots on tobacco leaves, YOLOX-Tiny does not meet the requirements in terms of detection accuracy and detection speed.

The basic structure of the YOLOX-Tiny network can be divided into input, backbone, neck, and prediction. The input part enriches the content augmentation of the dataset with mosaic data, and good detection results are achieved using low-cost computational resources. The backbone network consists of the focus module, the cross-stage partial (CSP) (Wang et al., 2020) layers, and the spatial pyramid pooling (SPP) module. The focus module slices the input image before the feature extraction, to increase the depth of the network and reduce the amount of computation in the network. YOLOX-Tiny can extract rich image features through the CSP layers, which are the residual edge of the convolutional layer, and then concatenate them with the main branch. The SPP module extracts image features at different scales by concatenating feature maps of different pooling layers. Path aggregation networks (PAN) (Wang et al., 2019) are used to aggregate the image feature in the neck network. Finally, two prediction heads are used in the prediction part, which performs the classification task and the regression task, respectively.

**Figure 3:**
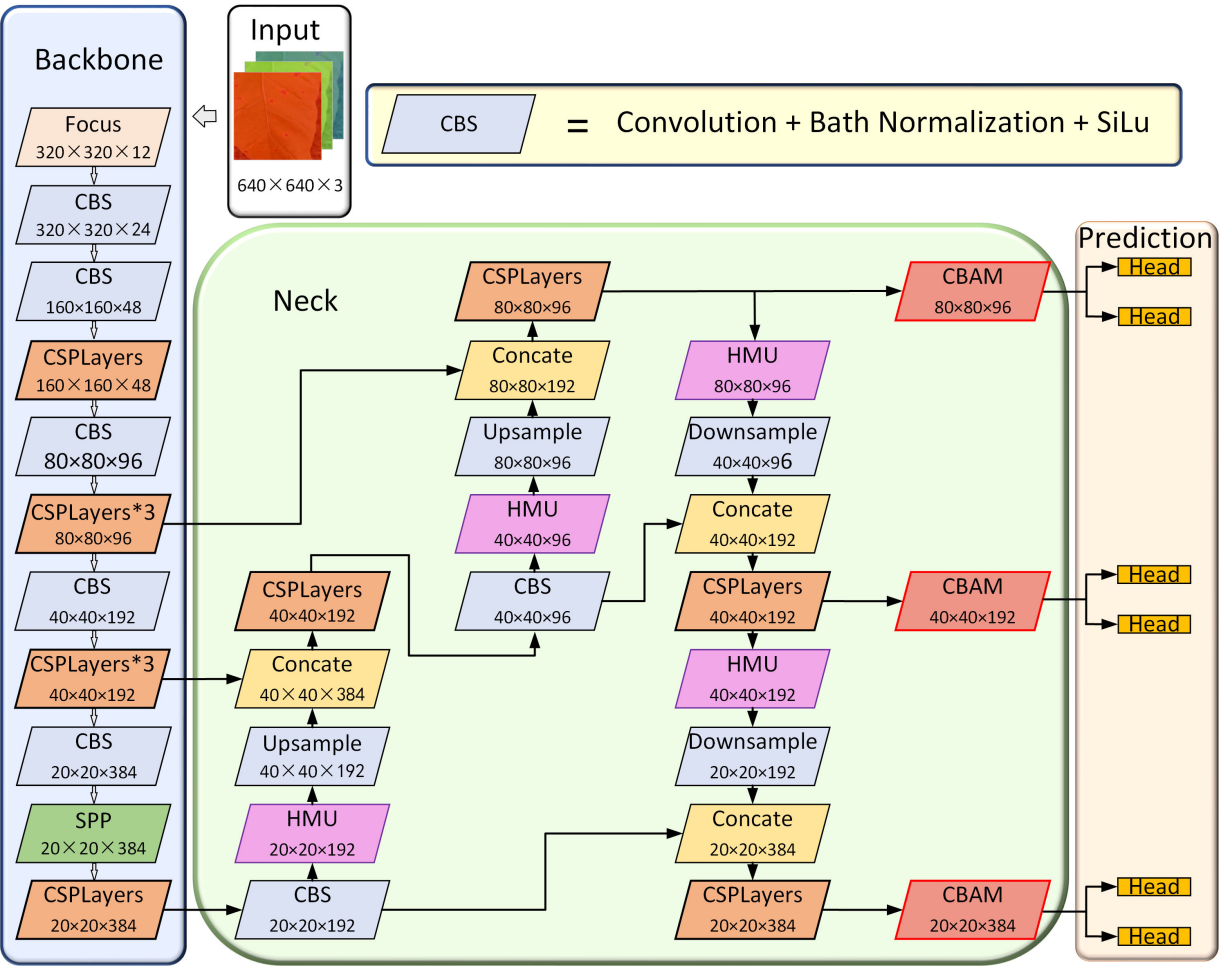
The architecture of the YOLO-Tobacco network. It consists of the input part, the backbone network part, the neck network part, and the prediction network part.

In this study, we introduced the HMU modules in the neck network. The HMU module can excavate important disease features, refine disease features in different channels, and enhance information interaction at different levels of the neck network, thus improving the detection performance of dense multi-scale disease spots. In addition, to enable the model to better focus on small objects and improve its robustness, we also introduced three CBAM modules before the prediction part. The architecture of the YOLOX-Tobacco network is shown in **Figure 3**.

### 3.2 HMU

**Figure 4:**
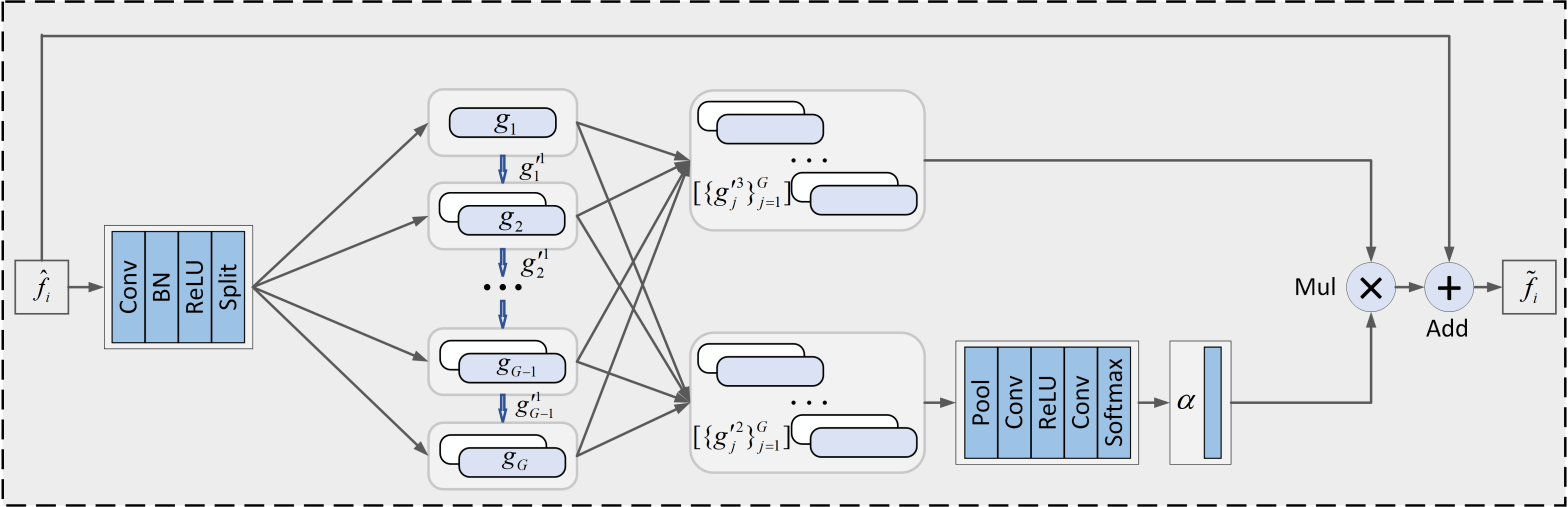
The structure of the HMU module.

Low-level features are suitable for detecting small objects, middle-level features are suitable for detecting medium-sized objects, and high-level features are suitable for detecting large objects. The original YOLOX-Tiny network incorporates features from different levels in the neck network through a top-down and bottom-up network structure. However, this plain approach did not extract the critical and refined information between each layer, making the model ineffective in detecting dense multi-scale disease spots. To do that, we used the HMU module in the neck network to conduct an effective fusion for the different levels of features. The structure of the HMU module is shown in **Figure 4**. It is composed of two parts, namely group-wise iteration and channel-wise modulation.

For the part of the group-wise iteration, given an input feature map $\hat{f}$ . First, a 1×1 convolution layer is used to increase the number of channels of the feature map and then divided it into *G* groups ${\left\{g_{j}\right\}}_{j=1}^{G}$ along the channel dimension. A convolution operation is used to divide the first group {*g_j_*}into three sets of features *α*. The first set of features ${\left\{{g^{\prime }}_{1}^{1}\right\}}_{k=1}^{3}$ interacts with the next group of features for feature interaction, while the other two sets of features are utilized for channel-wise modulation. For the *j*(1< *j*< *G*) group, the following operations performed are carried out: first, the feature map *g_j_* and the feature map ${g^{\prime }}_{j-1}^{1}$ are cascaded, then the feature map is subjected to convolution and split operations, and finally, the feature map is divided into three feature sets.

For the part of channel-wise modulation, first, the features $\left[{\left\{{g^{\prime }}_{j}^{2}\right\}}_{j=1}^{G}\right]$ yield the feature modulation vector *α* by a series of nonlinear operations. Then, the vector *α* is weighted as weights to the feature $\left[{\left\{{g^{\prime }}_{j}^{3}\right\}}_{j=1}^{G}\right]$ . Finally, the final output $\tilde{f}$ of the HMU module can be written as:


(1) $$\tilde{f}=A\left({\hat{f}}_{i}+N\left(T\left(\alpha \cdot \left[{\left\{g_{j}^{\prime }3\right\}}_{j=1}^{G}\right]\right)\right)\right)$$

Where *A* denotes the activation function, *N* denotes the batch normalization, and *T* denotes the convolution operation.

### 3.3 CBAM

**Figure 5:**
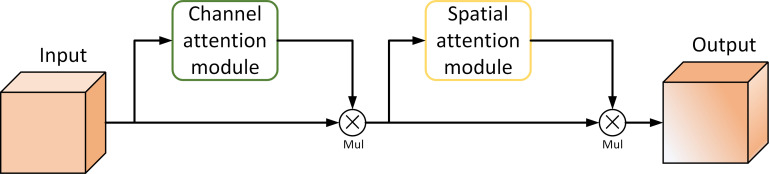
The structure of the CBAM module.

The CNN model can pay attention to valuable features and suppress invalid features by the attention mechanism. In this study, we used three CBAM modules in the neck network, thus making YOLOX-Tobacco pay more attention to key features and improving the detection ability of small disease spots. The structure of the CBAM module is shown in **Figure 5**. It aggregates important information first along the channel dimension and then along the spatial dimension. Therefore, the CBAM module can be divided into two modules, namely the channel attention module and the spatial attention module.

**Figure 6:**
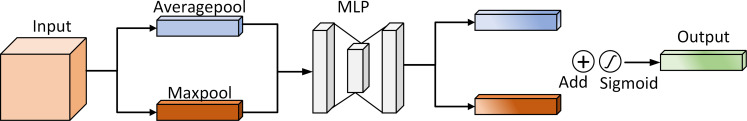
The structure of the channel attention module.


The structure of the channel attention module is shown in **Figure 6**. Given an input feature map *f* , we can get the output of the channel attention module:

(2) $$M_{c}(f)=\sigma (W_{1}(W_{0}\left(f_{\text{avg }}^{c}\right)+W_{1}\left(W_{0}\left(f_{\max}^{c}\right)\right)$$

Where *σ* represents the sigmoid activation function, The MPL weights (W_1_ and W_0_) are shared for both inputs and the ReLU activation function.

**Figure 7:**
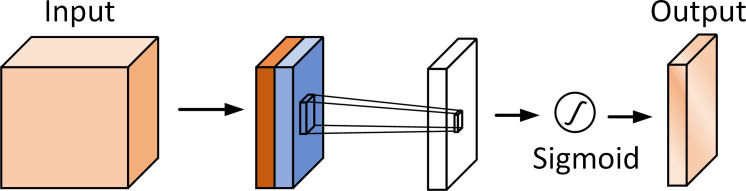
The structure of the spatial attention module.


The structure of the spatial attention module is shown in **Figure 7**. Given an input feature map *f*, the output of the spatial attention module can be written as:

(3) $$M_{c}(f)=\sigma \left(T^{7\times 7}\left(\text{ Concat }\left[f_{\text{avg }}^{c};f_{\max}^{c}\right]\right)\right)$$

Where *σ* represents the sigmoid activation function, *T* ^7×7^ represents a convolution layer with a filter size of 7.

## 4 Experimental results and analysis

### 4.1 Evaluation indexes

The precision, recall, F1-score, and average precision (AP) are used as evaluation indexes. The used formulas are as follows:


(4) $$Precision=\frac{TP}{TP+FP}$$

(5) $$Recall=\frac{TP}{TP+FN}$$

(6) $$F1-score=\frac{2TP}{2TP+FP+FN}$$

(7) $$AP=\int precision*recall=\int_{0}^{1}p\left(r\right)dr$$

Where *TP* is the number of true positive samples, *FP* is the number of false positive samples, *FN* is the number of false negative samples, and *TN* is the number of true negative samples. In addition, the frames per second (FPS) is also used to evaluate the performance of the model.

### 4.2 Experimental configuration and hyperparameter setting

The experimental configuration is shown in **Table 2**. The hyperparameters were set as follows. The stochastic gradient descent (SGD) optimizer was used to optimize the model. The initial learning rate was set to 0.01 with a momentum of 0.937 and a weight decay of 0.0005. We divided the training process into two stages. Specifically, in the first stage, we froze the weights of the backbone and trained the neck and prediction parts with training epochs of 50 and a batch size of 16. In the second stage, we trained all the parameters in the YOLOX-Tobacco network with training epochs of 100 and a batch size of 8. Remarkably, all networks used pre-trained weights of the backbone on the MS COCO dataset for transfer learning.

**Table 2:** Experimental configuration.

Name	Parameter
CPU	AMD Ryzen 9 5900X
GPU	NVIDIA GeForce RTX 3090
System	Windows 10
Programming Language	Python 3.7.13
Deep learning framework	Pytorch 1.12.1

### 4.3 Ablation experiment

We performed ablation experiments to verify the effect of the CBAM module and the HMU module on the original YOLOX-Tiny network. The results are shown in **Table 3**. The experimental results show that the YOLO-Tobacco network has a more comprehensive performance than the original YOLOX-Tiny network. Next, we analyzed each module in the YOLO-tobacco network.

**Table 3:** The results of ablation experiments with the confidence threshold is 0.5 and the IOU is 0.5.

YOLOX-Tiny	CBAM	HMU	AP (%)	Recall (%)	Precision (%)	F1-score	FPS
√			77.23	68.23	83.12	0.7494	79
√	√		79.29	69.90	83.98	0.7629	75
√		√	79.99	67.81	86.34	0.7596	74
√	√	√	80.45	69.27	86.25	0.7683	69

### 4.4 Performance comparison of each module

**Figure 8:**
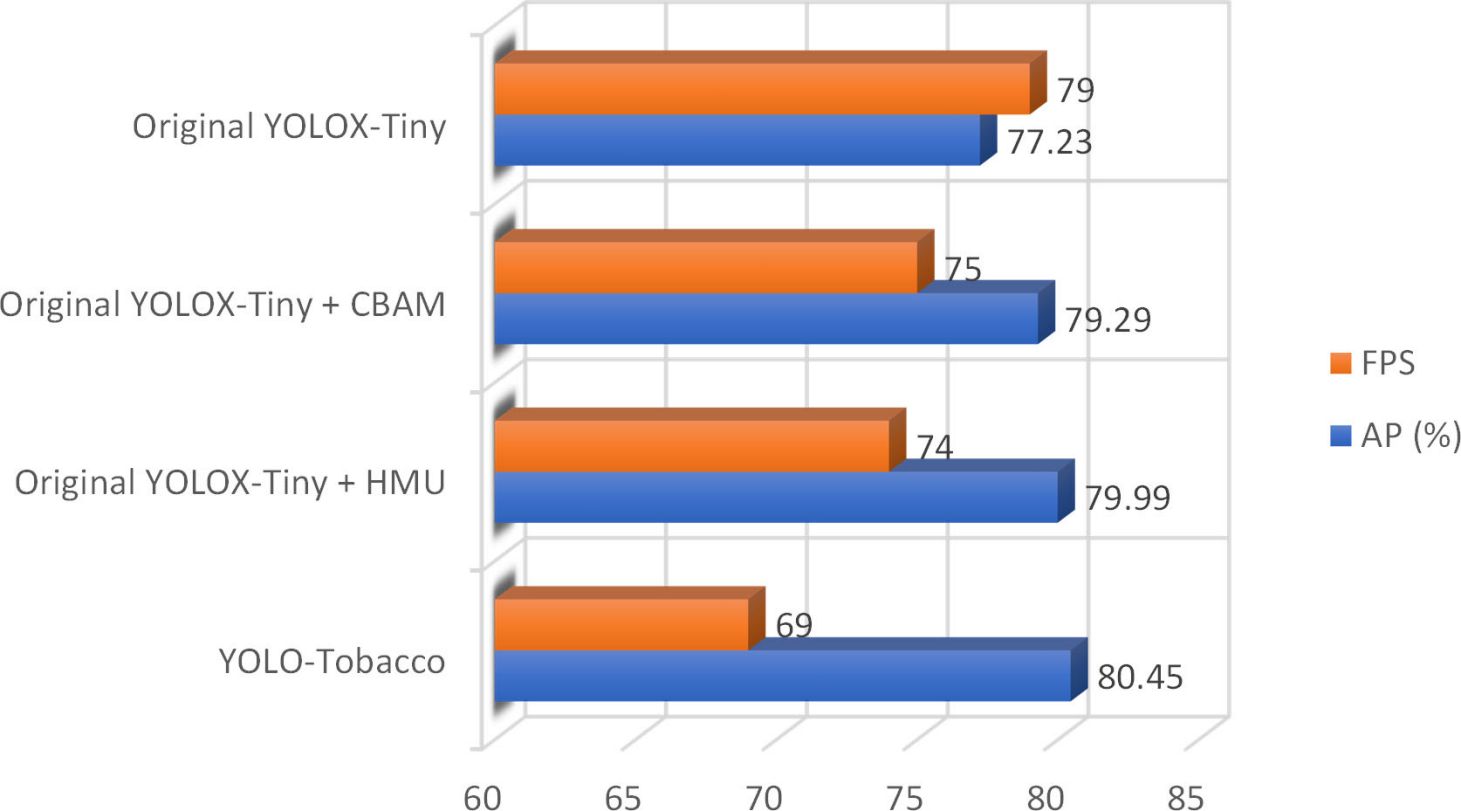
Comparison of the results of the four tested networks.

To visually demonstrate the impact of different modules on the network, we used a histogram to visualize two main metrics, namely AP and FPS. The comparison results of the four networks are shown in **Figure 8**. We found that the AP of the original YOLOX-Tiny network with the CBAM module reached 79.22%, which was 2.06% higher compared to the original YOLOX-Tiny network. This may be because the CBAM module can effectively reduce the influence of the complex background of tobacco leaves and improve the detection ability for small objects of tobacco brown spot disease. Subsequently, the original YOLOX-Tiny network with the HMU module achieved an AP of 79.99%, which was 2.76% higher than the original YOLOX-Tiny network. This may be because the HMU module emphasizes the critical information in the channel and enhances the fusion of different levels of features, thereby effectively improving the network’s ability to detect dense disease objects. Our proposed YOLO-Tobacco network, which contains both CBAM and HMU modules, achieved the highest detection accuracy with an AP of 80.45%, which was 3.22% higher than the original YOLOX-Tiny network. The FPS of the YOLO-Tobacco network was also reduced by 10. Overall, the experimental results demonstrate that the YOLO-Tobacco network achieves a better balance between detection accuracy and inference speed.

### 4.5 Comparison of the results with other tiny networks

To verify that the YOLO-Tobacco network has advantages over other tiny networks, we chose several common tiny YOLO networks to compare them with our proposed YOLO-Tobacco network. The comparison results are shown in **Table 4**. It can be seen that the AP, recall, precision, and F1-score of the original YOLOX-Tiny network showed substantial improvements compared to the YOLOv4-Tiny network and the YOLOv5-S network. The FPS of the original YOLOX-Tiny network was only about half that of the YOLOv4-Tiny network. This is because the structure of the YOLOv4-Tiny network is relatively simple and the number of detection heads is small, which improves its detection speed at the expense of detection accuracy. Afterward, compared to the three tiny networks, our proposed YOLO-Tobacco network achieved a state-of-the-art detection accuracy with 80.45% AP, 69.27% recall, 86.25% precision, and 0.7683 F1-score. Although the FPS of the YOLO-Tobacco network had a small decrease compared to the original YOLOX-Tiny network. Overall, the YOLO-Tobacco network achieved a trade-off between detection accuracy and detection speed.

**Table 4:** Comparison results of different tiny detection networks with the confidence threshold is 0.5 and the IOU is 0.5.

Networks	AP (%)	Recall (%)	Precision (%)	F1-score	FPS
YOLOv4-Tiny	68.42	54.17	80	0.6459	157
YOLOv5-S	71.46	58.33	80.23	0.6755	86
YOLOX-Tiny	77.23	68.23	83.12	0.7494	79
YOLO-Tobacco	80.45	69.27	86.25	0.7683	69

### 4.6 Analysis of the detection effect of different tiny networks

**Figure 9:**
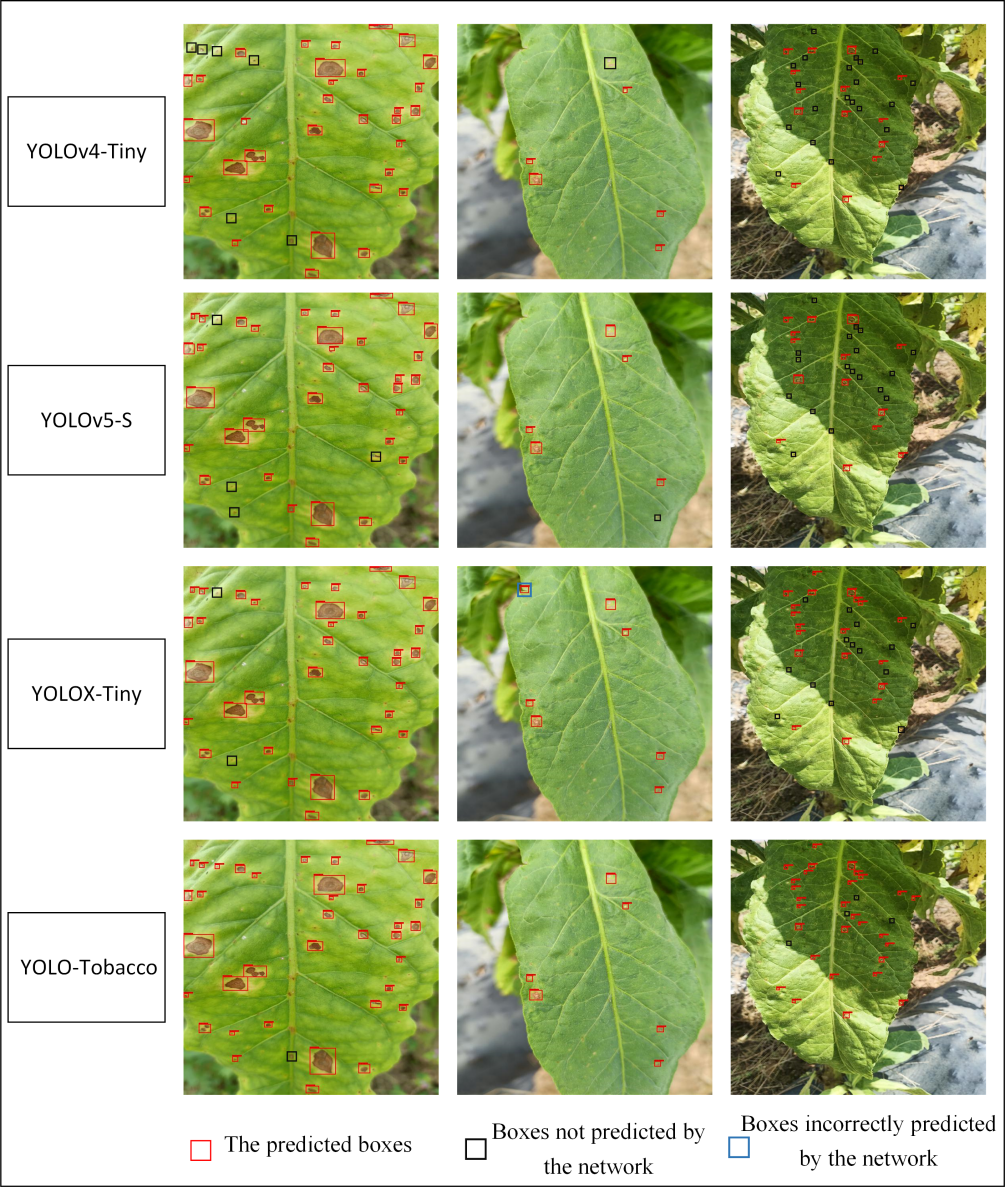
The comparison of different methods for the detection of brown spot disease.

For the detection of tobacco brown spot disease, the comparison of different networks (YOLOv4-Tiny, YOLOv5-S, YOLOX-Tiny, and YOLO-Tobacco) were shown in **Figure 9**. In the first column of images, the scale differences and dense distribution among the spots hamper the detection of spots. Our proposed YOLO-Tobacco network shows the best detection performance with the least number of missed spots.

In the second column of images, there are a small number of tiny spots, which are not obvious symptoms and therefore difficult to detect completely. Both the YOLOv4-Tiny network and the YOLOv5-S network had missed detection, although the YOLOX-Tiny network detected all targets but had false detection. YOLO-Tobacco network correctly detected all spots. The experimental results demonstrate that our proposed network has stronger feature discrimination performance.

In the third column of images, there are not only a large number of tiny spots but also uneven light distribution, which requires stronger robustness of the detection network. YOLOv4-Tiny, YOLOv5-S, and YOLOX-Tiny networks all had a large number of missed detections. However, the YOLO-Tobacco network only missed four spots. This demonstrates that the YOLO-Tobacco network shows more robustness in the presence of uneven illumination.

## 5 Conclusions

The classical object detection network showed insufficient performance in detecting images of tobacco brown spot disease with a large number of dense spots, inconspicuous features, and complex environments. Thus, we propose an improved object detection network, called YOLO-Tobacco, for detecting tobacco brown spot disease. By introducing HMU and CBAM modules into the original YOLOX-Tiny network, this method improves the ability of the network to extract features at different scales, refines discriminative features, and enhances the robustness of the model to solve the problems of dense distribution of disease spots in tobacco images, the inconsistent scale of disease spots, inconspicuous symptoms of disease spots, and network instability in complex condition. The experimental results demonstrate that the YOLO-Tobacco network outperforms existing lightweight networks in terms of detection accuracy, achieving an excellent balance between detection accuracy and detection speed. However, due to the limited number of datasets and the single plant disease used in this study, the proposed model still has spaces to be further optimized for improvement.

In the next future, we will collect more plant disease datasets of Solanaceae plants and develop further detection networks that can detect most leaf plant diseases of Solanaceae plant. Furthermore, optimizing works will be conducted to enhance the detection speed of the proposed network and deploy it to mobile or embedded devices to assist users quickly detect complex diseases of Solanaceae plant.

## Data availability statement

The datasets for this article are not publicly available because this research has been carried out as part of a project funded by the Government of China. Requests to access the datasets should be directed to XZ, xzhang1@gzu.edu.cn.

## Author contributions

XC and XZ designed the experiments. JWL, DY, RP, JC, JML, LZ, XW and XP performed the experiments. JWL, DY and RP analyzed data. JWL drafted the manuscript. XC, TC, SO, QM and XZ conducted visualization and proofreading of the manuscript. All authors approved the manuscript for submission.
